# Pathologic Response to Neoadjuvant Sequential Chemoradiation Therapy in Locally Advanced Breast Cancer: Preliminary, Translational Results from the French Neo-APBI-01 Trial

**DOI:** 10.3390/cancers15072030

**Published:** 2023-03-29

**Authors:** Nhu Hanh To, Isabelle Gabelle-Flandin, Thi My Hanh Luong, Gokoulakrichenane Loganadane, Nabila Ouidir, Chahrazed Boukhobza, Noémie Grellier, Camille Verry, Allan Thiolat, José L. Cohen, Nina Radosevic-Robin, Yazid Belkacemi

**Affiliations:** 1 Department of Radiation Oncology and The Henri Mondor Breast Center, Henri Mondor University Hospital, AP-HP, 1 Rue Gustave Eiffel, 94010 Creteil, France; nhuhanh.to@aphp.fr (N.H.T.);; 2 INSERM Unit 955, Immunoregulation and Biotherapy (I-Biot) Team, The Mondor Institute for Biomedical Research (IMRB), University of Paris-Est Creteil (UPEC), 94000 Creteil, France; 3 Transatlantic Radiation Oncology Network (TRONE), 94000 Creteil, France; 4 The Grenoble Alpes University Hospital Centre, University Clinic of Cancerology-Radiotherapy, Avenue des Maquis du Grésivaudan, 38041 Grenoble, France; 5 Solna Rheumatology Unit, Department of Medicine, Karolinska Institutet, 171 77 Solna, Sweden; 6 Department of Pathology, Henri Mondor University Hospital, AP-HP, 1 Rue Gustave Eiffel, 94010 Creteil, France; 7 INSERM Unit 1240 (IMoST), Radiopharmaceuticals & Biomarkers (RoBust) Team, Centre Jean Perrin, Department of Pathology, University Clermont Auvergne, 58 Rue Montalembert, 63000 Clermont-Ferrand, France

**Keywords:** neoadjuvant, radiotherapy, triple negative breast cancer, luminal B, neutrophils-to-lymphocytes ratio, tumor-infiltrating lymphocytes

## Abstract

**Simple Summary:**

Combined radiation therapy sequentially to neoadjuvant chemotherapy has been progressively assessed in breast cancer. The prediction of response to this innovative sequence is mandatory for treatment personalization. We conducted a translational study of the randomized phase 2 Neo-APBI-01 trial comparing standard neoadjuvant chemotherapy and neoadjuvant sequential chemoradiotherapy in locally advanced breast cancer of triple-negative and luminal B subtypes. Several potential immune-related biomarkers have been identified to be associated with pathologic complete response after chemoradiation therapy. The results of this study will constitute reflection support for the design of future trials evaluating the role of intact tumor-directed radiation therapy in breast cancers.

**Abstract:**

Background: Radiation therapy (RT), a novel approach to boost the anticancer immune response, has been progressively evaluated in the neoadjuvant setting in breast cancer (BC). Purpose: We aimed to evaluate immunity-related indicators of response to neoadjuvant chemoradiation therapy (NACRT) in BC for better treatment personalization. Patients and Methods: We analyzed data of the first 42 patients included in the randomized phase 2 Neo-APBI-01 trial comparing standard neoadjuvant chemotherapy (NACT) and NACRT regimen in locally advanced triple-negative (TN) and luminal B (LB) subtype BC. Clinicopathological parameters, blood counts and the derived parameters, total tumor-infiltrating lymphocytes (TILs) and their subpopulation, as well as *TP53* mutation status, were assessed as predictors of response. Results: Twenty-one patients were equally assigned to each group. The pathologic complete response (pCR) was 33% and 38% in the NACT and NACRT groups, respectively, with a dose-response effect. Only one LB tumor reached pCR after NACRT. Numerous parameters associated with response were identified, which differed according to the assigned treatment. In the NACRT group, baseline hemoglobin of ≥13 g/dL and body mass index of <26 were strongly associated with pCR. Higher baseline neutrophils-to-lymphocytes ratio, total TILs, and T-effector cell counts were favorable for pCR. Conclusion: This preliminary analysis identified LB and low-TIL tumors as poor responders to the NACRT protocol, which delivered RT after several cycles of chemotherapy. These findings will allow for amending the selection of patients for the trial and help better design future trials of NACRT in BC.

## 1. Introduction

Breast cancer (BC) management is a multimodal approach integrating surgery, radiation therapy (RT), and systemic treatment. The last decades have witnessed the emergence of new treatment paradigms, introducing neoadjuvant treatment (NAT) with more effective anticancer agents. Advances in knowledge of cancer immunoediting [1] and the cancer-immunity cycle [2] have enabled the development of modern cancer immunotherapy, with immune checkpoint inhibitors (ICIs), to reach an important inflection point in the history of cancer treatment. Recent studies evaluating the addition of an ICI (e.g., pembrolizumab or atezolizumab) have significantly increased the pathologic complete response (pCR) rate compared with neoadjuvant chemotherapy (NACT) alone in HER2-negative BC [3,4]. Novel combinations boosting antitumoral T-cell response are actively under investigation to overcome resistance to ICIs, such as platinum agents [5,6], cyclin-dependent kinase inhibitors [7], or RT [8].

Accelerated partial breast irradiation (APBI) has been increasingly incorporated in the management of early-stage BC in the adjuvant setting. Recent technical advances in RT have facilitated treatment delivery, especially when RT is directed to the tumor in the preoperative setting [9]. In addition, knowledge of distinct effects on the microvasculature and the immune system of higher doses of ionizing radiation (IR) has allowed the design of novel approaches to improve clinical outcomes in BC patients [8]. The neoadjuvant association of tumor-directed APBI and chemotherapy is expected to fully exploit the synergistic effect of radiation and cytotoxic agents with acceptable side effects. 

Only a few prospective studies have been published on RT responses of BC patients with intact tumors [10,11,12], as RT is traditionally given after breast surgery. An ongoing prospective study assessing neoadjuvant chemoradiation therapy (NACRT), the Neo-APBI-01 trial (NCT02806258), was specifically designed for aggressive HER2-negative locally advanced BC, i.e., triple negative (TN) and luminal B (LB) subtypes. Neo-APBI-01 aimed to exploit the ability of RT to induce immunogenic cell death, enhance the cancer-immunity cycle, and, therefore, improve the pCR rate of TN and LB tumors as a surrogate marker for better long-term outcomes [13,14]. Furthermore, the trial also provides a unique opportunity to assess in vivo the response of breast tumors and to evaluate potentially predictive biomarkers, helping to adjust the treatment individually. There is growing clinical evidence of the prognostic and predictive values of immunity/inflammation-related biomarkers, such as tumor-infiltrating lymphocytes (TILs) [15,16,17]. In addition, the patient’s systemic immune status, easily accessible by blood tests, has drawn attention as a promising and non-expensive biomarker of response to several anticancer therapies [18]. 

In this report, we present the results of the translational analysis of the Neo-APBI-01 trial, associated with an insight into potential predictive biomarkers. The conclusions we have drawn could be of consideration for the design of future NACRT trials in BC.

## 2. Materials and Methods

### 2.1. Patients

Between October 2016 and September 2021, forty-two patients were treated according to the Neo-APBI-01 protocol. The key eligibility criteria were: age ≥ 18 years, histologically proven invasive non-specific type or lobular breast carcinoma, and locally advanced disease of TN or LB/HER2- subtypes. 

**Figure 1:**
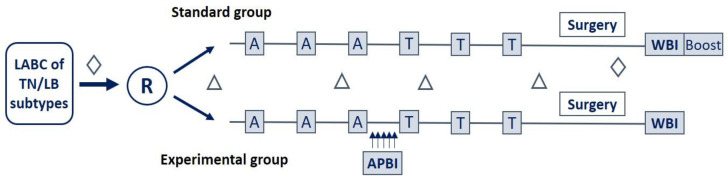
Schematic overview of the study. Patients were randomized to receive either standard treatment consisting of anthracycline/taxane-based NACT or experimental treatment with the same NACT and the addition of a short course tumor-directed APBI between two chemotherapy cycles. Abbreviation: A: anthracycline-based chemotherapy (FEC or EC: fluorouracil, epirubicin, cyclophosphamide), APBI: accelerated partial breast irradiation, LABC: locally advanced breast cancer, LB: luminal B, R: randomization, RT: radiation therapy, T: taxane, TN: triple-negative, WBI: whole breast irradiation (or parietal irradiation), ∆: blood test, ◊: histological analysis (biopsy before randomization and operative piece after breast surgery).

The patients were randomly assigned into two groups: the standard group received conventional anthracycline/taxane NACT. The number of cycles of the NACT regimen (3 or 4 cycles) and the choice of taxane molecule (paclitaxel or docetaxel) were left to the discretion of the local investigator. The experimental group had the same NACT regimen incorporating a sequential, short-course tumor-directed APBI. APBI was delivered between the penultimate cycle of the anthracycline-based regimen and the second cycle of taxane. Neoadjuvant RT consisted of 10 fractions of 2.5 Gray (Gy), two fractions per day (BID) over five days, or eight fractions of 3.125 Gy, one fraction per day (QID) over one week and a half. Breast surgery, lumpectomy or total mastectomy, and axillary lymph node dissection were performed four to six weeks after NAT completion. Postoperative RT was delivered according to the discretion of local physicians. No additional RT was added to the experimental NACRT group. Adjuvant hormonotherapy was prescribed for hormonal receptor-positive (HR+) patients (Figure 1).

Twenty-one patients were assigned to NACT only, and 21 received NACRT. Patients had consented to use their tumor tissue samples and blood test data for translational research analyses. The institutional review board approved the project (N°IDRCB: 2015-A01062-47).

### 2.2. Histopathological Assessments

Pretreatment histological type, grade, lymphovascular invasion, and molecular subtype by immunohistochemistry (IHC) were determined by local pathologists and reviewed by the central pathologist (NRR) for the study of biomarkers. The positivity for estrogen and progesterone receptors (ER and PR, respectively) was defined using the 10% cutoff. HER2 status was determined according to the ASCO/CAP criteria [19]. The molecular subtype was determined according to the Saint Gallen consensus 2013 [20]. 

pCR was defined as the absence of residual invasive cancer in the breast and the axillary lymph nodes (ypT0/Tis ypN0) without evidence of metastatic deposits [21]. 

IHC for p53, pRb, and PD-L1 was interpreted by the central pathologist as presented in Supplementary Table S1.

### 2.3. Assessment of Tumor-Infiltrating Lymphocytes (TILs)

The number of stromal TILs was assessed in H&E-stained tumor biopsies according to the International Immuno-Oncology Biomarker Working Group on Breast Cancer [22]. The tumors were classified into inflamed (IM), immune-excluded (IE), and immune-deserted (ID), according to Chen and Mellman [23].

TIL subpopulations expressing CD8, CD4, FoxP3, CD20, or PD-L1 were visualized by IHC performed as presented in Supplementary Table S1. QuPath software [24] was used to count stromal and intratumoral cells of each TIL subpopulation in a region of interest delineated to correspond to 10 consecutive high-power (×400) microscopic fields (field diameter 500 µm) from the invasive front to the tumor center. For each subpopulation, the number of cells per mm^2^ was calculated.

PD-L1 expression was expressed as the percentage of PD-L1+ tumor cells or PD-L1+ immune cells. 

### 2.4. Blood Cell Counts and the Derived Parameters

Blood tests were routinely performed at different time points (baseline, before and after APBI, and before surgery). We collected blood tests from different time points: (1) at baseline, within one week before the administration of any neoadjuvant treatment, (2) within two weeks before APBI delivery in the NACRT group, or two months after the initiation of NACT in the NACT group, (3) within two weeks after APBI delivery in the NACRT group, or three months after the initiation of NACT in the NACT group, (4) after NAT, collected within three weeks after the last cycle of NACT in both groups. The neutrophils-to-lymphocytes ratio (NLR) was calculated by dividing the absolute neutrophil count (ANC) by the absolute lymphocyte count (ALC). The platelets-to-lymphocytes ratio (PLR) was obtained by dividing absolute platelet count (APC) by ALC. The lymphocytes-to-monocytes ratio (LMR) was obtained by dividing the ALC by the absolute monocyte count. The systemic immune-inflammation index (SII) was defined as (ANC x APC)/ALC. The blood cell count-derived parameters dynamic changes (i.e., delta-NLR, delta-PLR, delta-LMR, and delta-SII) were defined as the post-NAT value minus the pre-NAT value of a given ratio. 

### 2.5. Statistical Analysis

The analysis was carried out on the first 42 patients included in the trial, triggered by the unusual behavior observed in some patients receiving NACRT [25]. The objective is to identify potential biomarkers predicting pCR after NACRT. The toxicity profile and survival outcomes will be reported separately at the closure of the study. Optimal biomarker cutoffs for pCR prediction were calculated considering the maximum (sensitivity and specificity) point of the Receiver Operating Characteristic (ROC) curve [26]. Associations between clinicopathological, blood cell-derived, or TIL-related parameters and response to NAT were evaluated by the Chi-squared test or Fisher’s exact test. Stromal TILs were assessed as continuous variables or predefined cutoffs of 10%, 30%, or 50%. Tumor or immune cell PD-L1 expression was evaluated as a binary variable, with 0%, 1%, and 10% cutoffs. Univariate analyses were performed using the logistic regression model. Multivariate analyses were not performed due to less than ten events in the analyzed cohorts [27]. The odds ratio (OR) was reported with the corresponding 95% confidence intervals (95% CI). The *p* values of <0.05 were considered statistically significant, and those of <0.1 and ≥0.05 as tendencies. Statistical analysis was performed using the R software, version 4.2.0 (R-Project, GNU GPL, https://cran.r-project.org).

## 3. Results

### 3.1. Patient and Tumor Characteristics

Patients and tumor baseline characteristics were comparable in both groups (Table 1). The median age at diagnosis was 48 years in the NACT group and 45 years in the NACRT group. The median tumor size was 30 mm (interquartile range, IQR: 25–38 mm) in the NACT group and 32 mm (IQR: 25–35 mm) in the NACRT group. The NACT group contained more patients with involved lymph nodes (*n* = 12, 57%) than the NACRT group (*n* = 8, 38%).

All patients presented with invasive carcinoma of non-specific type; most had histologic grade 3 and a very high Ki67 index. About one-third had an LB tumor, and two-thirds had a TNBC in both groups. 

Immunity-related biomarkers in the tumor microenvironment (TME) and blood in both groups were relatively similar. Supplementary Tables S2 and S3 present IHC and systemic inflammation markers, respectively. The immune deserted (ID) phenotype was most common, accounting for nearly half of the patients, corresponding to low TIL levels (<10%) in a majority of the tumors. About three-fourths of tumors had a *TP53* mutation (equally the null type and the missense type) and present pRb. Absolute blood cell counts and the derived ratios were also comparable in both groups, except for a slightly higher number of platelets in the NACRT group (median 309 G/L compared to 271 G/L in the NACT group, *p* = 0.05). Consequently, platelet-related ratios, i.e., PLR and SII, tended to be slightly higher in the NACRT group.

### 3.2. Response to Treatment

There was no statistically significant difference in the pCR rate between the NACRT and the NACT group (38% vs. 33%, respectively, *p* = 0.7). All but three pCR patients had a TNBC, and only one LB tumor responded to NACRT by pCR. Details of the response patterns are given in Table 2.

Among patients who received the total dose of 25 Gy, 43% of those who received 10 × 2.5 Gy BID (*n* = 6/14) and 67% of those who received 8 × 3.125 Gy QID (*n* = 2/3) reached a pCR, whereas no pCR was observed in patients receiving preoperative RT of 20 Gy (*n* = 4).

### 3.3. Associations of Putative Biomarkers and Response to Treatment

To evaluate the predictive capacity of clinical, blood, and tumor tissue parameters, we performed a logistic regression analysis. Globally, we observed that different parameters were associated with pCR in the NACRT cohort than in the NACT cohort.

Table 3 presents the associations between clinical parameters, peripheral blood counts, blood count-derived ratios, and pCR. In the cohort treated by NACRT, Hb level and body mass index (BMI) were the most significant parameters associated with pCR. None of the patients with Hb < 13 g/dL and BMI > 26 reached pCR. Furthermore, baseline NLR > 2.2 and delta-PLR > 3.3 G/L were associated with a higher probability of pCR. Finally, delta-NLR, baseline PNN, pre-APBI LMR, and the absence of involved LNs tended to correlate with pCR (Table 3). In the cohort treated by NACT, younger age (≤48 years) and a lower baseline SII (≤252) were favorable for pCR.

Table 4 presents the associations between tissular parameters and pCR. In the NACRT group, a very high Ki67 index (≥90%, *p* = 0.03) and higher tumor infiltration by T cells (the sum of CD8+ and CD4+ cells) (*p* = 0.05) were markedly associated with pCR. The TN subtype (*p* = 0.08), TILs ≥ 10% (*p* = 0.1), an immune phenotype other than the ID subtype (*p* = 0.1), and mutated *TP53* (*n* = 6/6) tended to correlate with pCR. On the other hand, a higher number of each assessed TIL subpopulation was associated with pCR in NACT-treated patients, as well as a higher ratio of CD8+ to FOXP3+ or CD8+ to CD4+ TILs. Patients with a higher expression of PD-L1 in immune cells (≥10%) tended to have a pCR to NACT.

## 4. Discussion

To our knowledge, this is the first study of a fractionated regimen of preoperative APBI associated with NACT to treat TN and LB tumors. Preoperative RT provides a unique opportunity to study radiation response and gain more insight into the radiation sensitivity of BC.

Only a few studies reported the outcomes of preoperative tumor-directed APBI in BC. Historically, APBI has been considered an option in selected patients of >50 years with HR+ early BC with a favorable prognosis [28]. In the neoadjuvant setting, one dose-escalating phase 1 study of tumor-directed APBI associated with taxane-based NACT reported an overall pCR rate of 36%, with a dose-dependent effect [10]. Table 5 summarizes data on prospective, tumor-directed APBI in the literature.

In our study, the pCR rate was 38% in the NACRT arm, similar to the pCR rates in previously published trials of chemo-RT in BC. A much higher pCR rate was observed in patients with a TN (54%) than with an LB tumor (13%). The TN subtype, harboring a higher proportion of lymphocyte-predominant tumors, has been well-known to respond more favorably to NACT [15,16]. Our study showed the same tendency of tumor response by subtypes; 38% and 25% of TN and LB patients reached a pCR after NACT, respectively.

By evaluating clinicopathological and systemic immune parameters, we identified several factors associated with response, which differed according to the assigned treatment. In the NACRT group, the absence of anemia, a normal BMI, and systemic inflammation-related index are markedly observed in pCR patients. In contrast, well-established favorable factors for a better response to NACT, such as young age, a higher TIL, and TIL subpopulations, were correlated to pCR.

The parameters strongly associated with pCR in the NACRT cohort were Hb ≥ 13 g/dL and BMI < 26. Tissue oxygenation is a key component modulating responsiveness to ionizing radiation. Accumulating evidence has strongly suggested that poor intratumoral oxygenation and the presence of anemia can adversely influence the survival of numerous cancer patients [32] and outcomes following curative-intent RT [33]. The negative impact of pretreatment anemia (using a Hb level threshold of 9–14.5 g/dL) on locoregional control and survival has been consistently documented in patients with various solid tumors (e.g., head and neck, uterine cervix, lung, anus, and prostate) [33]. All patients in our study obtained a pCR after NACRT had the pretreatment Hb level of ≥13 g/dL. Adequate oxygenation has recently been demonstrated to suppress immunosuppressive metabolites and cytokines in the TME and confer a favorable immunity-mediated response to treatment [34].

Other immunity-related factors could also alter the response to anticancer therapy. Excessive body fatness is linked to systemic and intratumoral chronic and TME inflammation, which diminishes the anticancer immune response. Inflammation is considered a hallmark of cancer establishment and progression [35]. It has been reported that overweight and obese patients (defined as BMI ≥25 and 30, respectively) experienced reduced efficacy and increased anticancer treatment-related toxicities, as well as inferior outcomes [36]. These patients also had a lower pCR rate when treated by NACT compared to those with under-/normal weight [37,38]. In line with these findings, all the patients who reached a pCR after NACRT in our study had normal weight. Maintaining a healthy weight is an important topic in patients’ lifestyle education, helping improve the quality of life and treatment outcomes.

In contrast with most published studies on NACT in BC, high pretreatment NLR (>2.2) was favorable for pCR in our NACRT-treated cohort. Neutrophils are the most abundant leukocytes in the blood and are emerging as important regulators of cancer [39]. Elevated counts of neutrophils and a neutrophil-derived parameter NLR were found in advanced malignant disease and considered an indicator of poor prognosis in several cancers [40]. Recent research has revealed the multifaceted roles of neutrophils; in some contexts, these cells may exert strong antitumoral activities [39]. Those findings might explain the controversies about NLR as a prognostic and predictive indicator in different cancers, including BC [40,41,42]. RT to the intact breast tumors produces inflammatory cytokines such as TNF-α, IFN-γ which can promote neutrophil differentiation towards the N1 antitumor phenotype, which might explain why more pretreatment neutrophils can be favorable for tumor elimination by a NACRT [39].

Although the level of statistical significance was not reached, likely due to the small number of patients, at least 10% TIL and higher numbers of T effector cells (CD8+ and CD4+) were favorable for pCR to NACRT in our study. These associations were more significant in the NACT group and in concordance with the literature [43]. An immune-effective TME was necessary for the survival benefit of RT in BC [44]. Placing preoperative APBI in the middle of NACT when the immune TME was impoverished might have been a reason why the immunogenic antitumoral effect of RT was not observed in the study. The lack of the immunity-boosting effect of RT could have been even greater in the LB than in the TN tumors, as the former typically contained less TIL than the latter [15]. Interestingly, the only LB tumor that responded by pCR in our NACRT cohort was much richer in TILs than the other tumors of this subtype [45]. A fraction of TIL-rich BCs, greater in the TN than in the LB subtype, have defects in the DNA repair pathway and are more chemo/radiosensitive. This intrinsic tumor cell characteristic is likely a major biomarker of response to any DNA-damaging therapy, including RT, so it should be assessed by dedicated tests in future trials of NACRT in BC.

Ki-67 is a biomarker of cell proliferation. The International Ki-67 in BC Working Group has validated Ki-67 as a prognostic marker in BC. However, they concluded that its clinical utility remains limited to prognosis assessment in early-stage (stage I or II) ER+/HER2- BC [46]. Many studies also suggested the association between a higher Ki-67 index and unfavorable tumor characteristics and poorer outcomes, as well as its value in improving the prediction of systemic treatment response [47]. Our study showed that tumors with a very high Ki-67 index responded more favorably to NACRT, depicting the intrinsic radiosensitivity of rapidly-growing tumors. Another study also reported the Ki-67 index of ≥80% to be predictive of complete response in small cell lung cancer patients treated with concurrent or sequential chemoradiotherapy [48].

Related to tumor DNA repair, an interesting observation in this study was that no tumor without mutated *TP53* reached pCR to NACRT. The only LB tumor which had a pCR was also *TP53*-mutated*. Most of these tumors had a missense *TP53* mutation*. None of the *TP53*-mutated tumors had the ID phenotype, whereas among the ID tumors (all non-pCR), only one was *TP53*-mutated* [45]. The association between the missense *TP53* mutation and higher TIL levels has been recently described [49]. In the LB subtype, *TP53* mutation is associated with a more aggressive disease [50] and a higher frequency of other mutated genes involved in DNA repair [51]. Therefore, screening for *TP53* mutations by IHC might give an insight into cancer chemo/radiosensitivity and help better select BC patients for NACRT approaches.

The pRb status, assessed by IHC, was not associated with response in this small cohort of patients. However, the only patient with a null-type *TP53* mutation and absent pRb (corresponding to the p53 loss/pRb loss status) among the 21 treated by NACRT was primo-resistant to the treatment and experienced a rapid fatal metastatic relapse [25]. As in the case of *TP53* mutations, an IHC-based assessment of the pRb status seems worth further evaluation in the NACRT trials. 

Several limitations are worth noting in our present study. Notably, the low number of included patients and the low number of complete responders make it insufficient to detect significant and clinically relevant differences in potential predictive factors. Blood tests were performed according to patients’ convenience in different laboratories, and inter-laboratory and inter-individual differences might exist. Despite these limitations, this is, to our knowledge, the first cohort of NACRT-receiving patients in which immunity-related potential predictive biomarkers were evaluated.

## 5. Conclusions

This preliminary analysis of the association of NACRT with a short course of tumor-directed APBI revealed the poor response of LB and low-TIL tumors to a regimen having a low-dose RT placed after several cycles of chemotherapy. These findings will allow us, in the planned amendment of the Neo-APBI-01 trial, to adapt the selection criteria by excluding patients with LB tumors. Moreover, this will constitute reflection support for the design of future trials. Finally, each NACRT will likely have specific predictive biomarkers; therefore, translational studies associated with NACRT trials should be deeply explorative.

## Figures and Tables

**Table 1:** Standard clinical and histological characteristics of the analyzed cohorts.

Variable	N	NACT, N = 21	NACRT, N = 21	*p*-Value ^1^
**Age** (y) Median (IQR)	42	48 (41, 52)	45 (40, 50)	0.3
**Menopause**	42			>0.9
No		16 (76%)	17 (81%)	
Yes		5 (24%)	4 (19%)	
**Body Mass Index**	42			0.5
Normal		10 (48%)	12 (57%)	
High		11 (52%)	9 (43%)	
**Tumor size**	42			0.2
cT 1		3 (14%)	0 (0%)	
cT 2		15 (71%)	19 (90%)	
cT 3		3 (14%)	2 (9.5%)	
**Nodal status**	42			0.5
cN 0		9 (43%)	13 (62%)	
cN +		12 (57%)	8 (38%)	
**Histological grade**	42			0.5
Grade 2		7 (33%)	5 (24%)	
Grade 3		14 (67%)	16 (76%)	
**Ki67 Median (IQR)**	42	80 (60, 95)	85 (60, 95)	>0.9
**Molecular subtype**	42			>0.9
Triple-negative		13 (62%)	13 (62%)	
Luminal B		8 (38%)	8 (38%)	

^1^ Wilcoxon rank sum exact test; Fisher’s exact test; Pearson’s Chi-squared test; Wilcoxon rank sum test. Legend: cN: clinical nodal status (+: involved), cT: clinical tumor status, IQR: interquartile range, NACT: neoadjuvant chemotherapy, NACRT: neoadjuvant chemo-radiotherapy.

**Table 2:** Response to neoadjuvant therapy.

Response	NACT, N = 21	NACRT, N = 21
**pCR, *n* (%)**	**N = 7 (33%)**	**N = 8 (38%)**
Triple-negative	5 (71%)	7 (88%)
Luminal B	2 (29%)	1 (12%)
**Response to neoadjuvant therapy**		
Any primary tumor downstaging	20 (95.3%)	20 (95.3%)
Mean tumor size reduction (mm)	26.6	23.0
cN− to ypN−	8 (38.1%)	12 (57.1%)
cN+ to ypN−	9 (42.9%)	2 (16.7%)
cN− to ypN+	1 (4.8%)	1 (4.8%)
cN+ to ypN+	3 (14.3%)	6 (28.6%)

Legend: cN: clinical node classification, NACT: neoadjuvant chemotherapy, NACRT: neo-adjuvant chemoradiation therapy, pCR: pathologic complete response, ypN: pathologic nodal stage after neoadjuvant treatment.

**Table 3:** Associations between clinical and peripheral blood parameters and response to neoadjuvant treatment.

			NACT				NACRT	
Parameters	pCR+	pCR−	OR (95% CI)	*p*-Value	pCR+	pCR−	OR (95% CI)	*p*-Value
**Age (years)**				**0.004**				0.52
≤48	7	4	—		6	8	—	
>48	0	10	0		2	5	0.53 (0.1–3.5)	
**Body mass index**				>0.99				**0.018**
≤26	4	8	—		8	6	—	
>26	3	6	1 (0.2–6.3)		0	7	0	
**LN involvement**				>0.99				***0.08***
Negative	3	6	—		7	6	—	
Positive	4	8	1 (0.1–6.8)		1	7	0.1 (0.1–1.0)	
**Baseline PNN**				0.34				***0.08***
<3.3 G/L	5	7	—		1	7	—	
≥3.3 G/L	2	7	0.4 (0.1–2.6)		7	6	8.2 (1–177)	
**Baseline Hb**				0.34				**0.006**
<13 g/dL	5	7	—		0	8	—	
≥13 g/dL	2	7	0.4 (0.1–2.6)		8	5	8.2 (1–177)	
**Baseline NLR**				>0.99				**0.03**
≤2.2	5	9	—		3	10	—	
>2.2	2	5	1 (0.1–7.2)		5	3	10 (1.5–101)	
**Baseline SII**				**0.05**				0.15
≤252	4	7	—		0	2	—	
>252	3	7	0.1 (0.01–1)		8	11	3 × 10^7^ (0–NA)	
**Pre-APBI LMR**				>0.99				***0.08***
<1.9	3	7	—		2	9	—	
≥1.9	4	7	1.3 (0.2–9)		6	4	8.2 (1–177)	
**Delta-NLR**				>0.99				***0.06***
<0.8	4	7	—		2	7	—	
≥0.8	3	7	1 (0.2–6.4)		6	6	6.8 (1–63)	
**Delta-PLR**				0.74				**0.05**
<120	2	5	—		2	8	—	
≥120	5	9	1.4 (0.2–12)		6	5	11 (1.4–245)	

Legend: CI: Confidence Interval, Hb: hemoglobin, LMR: lymphocytes-to-monocytes ratio, LN: lymph node, NA: not applied, NACT: neoadjuvant chemotherapy, NACRT: neoadjuvant chemo-radiotherapy, NLR: neutrophils-to-lymphocytes ratio, OR: Odds Ratio, PLR: platelets-to-lymphocytes ratio, pCR: pathologic complete response (+: yes, −: no), PNN: polymorphonuclear neutrophils, SII: systemic immune inflammation index.

**Table 4:** Associations between tissular parameters and response to neoadjuvant treatment.

			NACT				NACRT	
Parameters	pCR+	pCR−	OR (95% CI)	*p*-Value	pCR+	pCR−	OR (95% CI)	*p*-Value
**Subtype**				0.52				***0.08***
Triple-negative	5	8	—		7	6	—	
Luminal B	2	6	0.5 (0.1–3.5)		**1**	7	0.1 (0.1–1.0)	
**Ki-67 index**				0.54				**0.03**
<90%	3	8	—		2	10	—	
≥90%	4	6	1. 8 (0.3–12)		6	3	10 (1.5–101)	
**TILs (%)**				0.68				***0.1***
<10	4	8	—		3	8	—	
≥10	3	4	1.5 (0.2–11)		5	5	4.8 (0.8–43)	
**ID phenotype**				0.87				***0.1***
No	5	3	—		**8**	8	—	
Yes	2	9	1.2 (0.1–9.9)		**0**	4	0	
**TIL-CD8+**				**0.01**				0.25
<930/mm^2^	2	11	—		4	9	—	
≥930/mm^2^	5	1	28 (2.8–725)		4	3	3 (0.5–23)	
**TIL-CD4+**				***0.08***				0.36
<1360/mm^2^	2	9	—		3	8	—	
≥1360/mm^2^	5	3	6 (0.8–64)		5	4	2.3 (0.4–16)	
**TIL-FOXP3+**				**0.04**				0.55
<480/mm^2^	3	11	—		5	9	—	
≥480/mm^2^	4	1	15 (1.5–356)		3	3	1.8 (0.3–13)	
**TIL-CD20+**				**0.01**				0.36
<428/mm^2^	1	11	—		3	7	—	
≥428/mm^2^	6	1	66 (5–2,648)		5	5	2.3 (0.4–16)	
**TIL-T cells**				1				**0.05**
<3076/mm^2^	3	12	—		3	10	—	
≥3076/mm^2^	4	0	4 × 10^8^ (0–NA)		5	2	8.3 (1.2–86)	
**TIL-CD8/FOXP3**				**0.02**				0.46
<1.9	2	9	—		4	4	—	
≥1.9	5	3	18 (2–426)		4	8	0.5 (0.1–3.1)	
**TIL-CD8/CD4**				**0.03**				0.46
<0.4	2	9	—		4	4	—	
≥0.4	4	3	15 (1.6–360)		4	8	0.5 (0.1–3.1)	
**PD-L1 ic**				***0.07***				0.51
<10%	3	11	—		5	5	—	
≥10%	3	1	11 (1–276)		2	4	0.5 (0.1–3.9)	
***TP53* mutation**				0.68				0.2
No	1	3	—		**0**	4	—	
Yes	6	9	1.5 (0.2–11)		**6**	7	9 × 10^7^ (0–NA)	

Legend: CI: Confidence Interval, ID: immune-deserted, NA: not applied, NACT: neoadjuvant chemotherapy, NACRT: neoadjuvant chemo-radiotherapy, OR: Odds Ratio, pCR: pathologic complete response (+: yes, −: no), PD-L1 ic: programmed death-ligand 1 presented on immune cells, TIL: tumor-infiltrating lymphocyte.

**Table 5:** Prospective studies assessing preoperative, tumor-directed APBI.

Author, Year [Ref.]	N	Tumor Characteristics	RT Dose	NACT	Time to Surgery	pCR Rate
Bondiau et al., 2013 [10]	25	Unifocal, HER2-, BCS unsuitable	3 × 6.5–10.5 Gy	**Yes**	4–8 weeks	**36%**
Van der Leij et al., 2015 [29]	70	>60 yo, unifocal ≤ 3 cm, SLN-	10 × 4 Gy or 5 × 6 Gy	No	6 weeks	10% (near-pCR)
Nichols et al., 2017 [12]	27	Unifocal, <3 cm, cN0	10 × 3.85 Gy bid	No	21 days	15%
Horton et al., 2018 [30]	32	≥55 yo, T1, cN0, ER/PR+ HER-	1 × 15–21 Gy	No	10 days	NR
Yaremko et al., 2018 [31]	39	Unifocal, <3 cm, cN0, ER+	1 × 21 Gy	No	1 week	NR
Current study	21	Locally advanced TNBC, LB	10 × 2.5 Gy bid or 8 × 3.125 Gy qid	**Yes**	4–6 weeks	**38%**

Legend: BCS: breast-conserving surgery, bid: twice a day, ER: estrogen receptor, HER2: human epidermal growth factor receptor 2, ICI: immune checkpoint inhibitor (pembrolizumab), LB: luminal B, mTNBC: metastatic triple-negative breast cancer, N: number of patients, NA: not applied, NACT: neoadjuvant chemotherapy, NR: not reported, ORR: overall response rate, pCR: pathologic complete response, PR: progesterone receptor, qid: once a day, SLN: sentinel lymph node, yo: years old.

## Data Availability

Research data are stored in an institutional repository and will be shared upon request to the corresponding author.

## Supplementary Materials

The following supporting information can be downloaded at: https://www.mdpi.com/article/10.3390/cancers15072030/s1, Reference [52] is cited in the supplementary materials. Table S1: Immunohistochemical staining; Table S2: Immune-related histological markers; Table S3: Immune-related systemic markers.
