# Supplementary material for: Pathologic Response to Neoadjuvant Sequential Chemoradiation Therapy in Locally Advanced Breast Cancer: Preliminary, Translational Results from the French Neo-APBI-01 Trial
Source: Cancers (Basel). 2023 Mar 29;15(7):2030. doi: 10.3390/cancers15072030 (PMC10092968; doi:10.3390/cancers15072030)
Supplement: Supplementary file 1 [file cancers-15-02030-s001.zip › cancers-2283847-supplementary.pdf]

Supplementary Table S1: Immunohistochemical staining

Antigen	Clone	Distributor	Dilution	Incubation	Detection	Interpretation
**p53**	DO-7	Agilent	1/200	40 min	ultraView DAB	According to [52].<br>Strong diffuse nuclear staining in tumor cells: TP53 missense mutation(s)<br>No nuclear staining in tumor cells with heterogeneous moderate/weak staining in benign cells: TP53 'null' mutation<br>Heterogeneous moderate/weak nuclear staining in tumor cells: no TP53 mutation
**pRb**	358	Novocastra	1/50	60 min	OptiView DAB	pRb present: tumor cell nuclear staining*
**CD8**	SP16	ThermoFisher Scientific	1/200	60 min	OptiView DAB	CD8+ TILs: with membranous staining*
**CD4**	SP35	CellMarque	1/50	60 min	ultraView DAB	CD4+ TILs: with membranous staining*
**CD20**	SP32	CellMarque	1/200	60 min	ultraView DAB	CD20+ TILs: with membranous staining*
**FoxP3**	SP97	ThermoFisher Scientific	1/100	60 min	ultraView DAB	FoxP3+ TILs: with nuclear staining*
**PD-L1**	28-8	Abcam	1/50	60 min	OptiView DAB	% of tumor cells with membranous staining*<br>% of immune cells (TILs and macrophages) with membranous staining*

*Legend: ultraView DAB, ultraView Universal DAB Detection Kit (Ventana/Roche, Meylan, France); OptiView DAB, OptiView DAB IHC Detection Kit (Ventana/Roche, Meylan, France); *any intensity; TILs, tumor-infiltrating lymphocytes. All stainings were performed in Benchmark Ultra automates (Ventana/Roche, Meylan, France).*

Supplementary Table S2: Immune-related histological markers

**Variable**	**N**	**NACT**, N = 21[1]	**NACRT**, N = 21[1]	**p-value**[2]
**Immuno-phenotype**	39			0.8
Inflamed		5 (26%)	7 (35%)	
Excluded		9 (47%)	9 (45%)	
Deserted		5 (26%)	4 (20%)	
**Tumor-infiltrating lymphocytes**	40			0.5
< 10%		12 (63%)	10 (48%)	
≥10% - <30%		4 (21%)	7 (33%)	
≥30% - <50%		0 (0%)	2 (9.5%)	
≥50%		3 (16%)	2 (9.5%)	
**TIL subpopulations (per mm²)**				
TIL CD8+	39	399 (235, 1,097)	583 (269, 1,069)	0.6
TIL CD4+	38	1,227 (595, 1,577)	1,255 (855, 2,099)	0.4
TIL FOXP3+	39	234 (95, 445)	331 (140, 562)	0.5
TIL CD20+	39	226 (53, 604)	446 (216, 1,238)	0.2
TIL T lymphocytes	39	1,502 (770, 2,420)	2,011 (1,020, 3,365)	0.4
TIL total lymphocytes	39	1,902 (1,002, 2,819)	2,502 (1,249, 4,603)	0.5
***TP53* mutation status**	36			>0.9
No mutation		4 (21%)	4 (24%)	
Mutated type null		8 (42%)	6 (35%)	
Mutated type missense		7 (37%)	7 (41%)	
**pRb status**	36			>0.9
Absent		5 (26%)	4 (24%)	
Present		14 (74%)	13 (76%)	

[1] n (%); Median (IQR)
[2] Fisher's exact test; Wilcoxon rank sum exact test

Legend: NACT: neoadjuvant chemotherapy, NACRT: neoadjuvant chemo-radiotherapy, TIL: tumor-infiltrating lymphocyte.

Supplementary Table S3: Immune-related systemic markers

Variable	N	**NACT**, N = 21[1]	**NACRT**, N = 21[1]	p-value[2]
**Leukocyte (G/L)**	42	5.80 (4.60, 6.70)	6.60 (5.70, 7.84)	0.2
**Neutrophil (G/L)**	42	3.06 (2.10, 4.22)	3.94 (2.92, 5.24)	0.11
**lymphocyte (G/L)**	42	1.70 (1.57, 2.48)	1.83 (1.41, 2.35)	>0.9
**Monocyte (G/L)**	42	0.48 (0.34, 0.50)	0.50 (0.40, 0.62)	0.2
**Platelet (G/L)**	42	271 (222, 301)	309 (265, 361)	**0.05**
**Hemoglobin (g/dL)**	42	12.90 (12.70, 13.60)	13.40 (12.50, 13.90)	0.5
**NLR**	42	1.77 (1.02, 2.24)	1.77 (1.22, 3.29)	0.3
**PLR**	42	149 (109, 186)	201 (125, 211)	***0.10***
**LMR**	42	4.00 (3.31, 4.71)	3.55 (3.13, 4.61)	0.4
**SII**	42	513 (253, 825)	650 (378, 1,018)	***0.087***

[1] Median (IQR)

[2] Wilcoxon rank sum test; Wilcoxon rank sum exact test

Legend: LMR: lymphocyte-monocyte, NACT: neoadjuvant chemotherapy, NACRT: neoadjuvant chemo-radiotherapy, NLR: neutrophil-lymphocyte ratio, PLR: platelet-lymphocyte ratio, SII: systemic immune-inflammation index.
